# Color-Blindness

**Published:** 1853-08

**Authors:** 


*Color-Blindness.*—Dr. Wilson, of Edinburgh, in a paper in the *Athenæum*, refers to this subject as having an important bearing on the system of railway signalling by colors' especially by red and green danger signals. This affection ("Daltonism," *Cromato pseudopsis, Achromatopsia,* etc.,) Dr. Wilson considers in three important practical relations; 1. That the affection is much more prevalent than is generally imagined. 2. That red and green, the colors used for danger signals on our railways, are exactly those which are most frequently confounded with each other by the subjects of color-blindness. 3. That color-blindness implies not merely a confusion in distinguishing between two or more colors, but, at least in many cases, an imperfect appreciation or feeble hold of color altogether as a quality of bodies. Prevost says, that color-blindness occurs in one male among twenty. Seebeck (in Poggendorff, Annalen, xliii. 177) found five cases among forty youths in Berlin. Professor Kelland, of the University of Edinburgh, has this session found three examples among 150 students. In four of the cases which had come under Dr. Wilson's notice, none of them could be trusted to distinguish a red signal from a green one , and there was nct only false vision of colors, but, in many instances, total color-blindness—so that the subjects of it doubted as to all colors, and would not swear in a court of justice as to any color. These facts, in connection with the continually augmenting number of railway accidents occuring, must show the imperative necessity of strict examination being instituted as to the perfect vision of all railway servants.—*Med. Gazette.*

